# The efficacy and the safety of eltrombopag in pediatric patients with severe aplastic anemia: a systematic review

**DOI:** 10.3389/fped.2023.1149718

**Published:** 2023-04-24

**Authors:** Maria Maddalena Marrapodi, Annamaria Mascolo, Domenico Roberti, Martina Di Martino, Concetta Rafaniello, Consiglia Riccardi, Francesca Rossi

**Affiliations:** ^1^ Department of Woman, Child and General and Specialist Surgery, University of Campania “Luigi Vanvitelli”, Naples, Italy; ^2^ Campania Regional Centre for Pharmacovigilance and Pharmacoepidemiology, University of Campania “Luigi Vanvitelli”, Naples, Italy; ^3^ Department of Experimental Medicine—Section of Pharmacology “L. Donatelli”, University of Campania “Luigi Vanvitelli”, Naples, Italy

**Keywords:** eltrombopag, severe aplastic anemia, children, safety, efficacy

## Abstract

**Background:**

Acquired aplastic anemia (AAA) in pediatric patients is a rare disorder characterized by hypocellular bone marrow and pancytopenia. Eltrombopag, an oral thrombopoietin receptor agonist, provides a hematologic improvement in adults with severe aplastic anemia (SAA) refractory to immunosuppressive therapy (IST). The association of ELT and IST was approved by the US Food and Drug Administration (FDA) for adults and children ≥2 years of age as a first-line treatment for SAA. However, the effects of ELT on pediatric patients with SAA remain controversial and limited.

**Methods and findings:**

We conducted a systematic review of the most recent literature from Pubmed, Web of Science, and Embase, published up to 20th December 2022, in order to evaluate the available evidence on the efficacy and safety of ELT added to IST for the treatment of SAA in the pediatric population.

**Conclusion:**

Eltrombopag added to the IST has shown a good safety profile, without manifestations of excessive toxic effects, although not all the results obtained from our studies support the addition of ELT to the IST in the first-line treatment of children with SAA.

**Systematic Review Registration:**

https://www.crd.york.ac.uk/prospero/, identifier: CRD42022325859.

## 1. Introduction

Severe Aplastic Anemia (SAA) is an acquired bone marrow disease characterized by a shortage of hematopoietic stem and progenitor cells, and trilinear marrow hypoplasia (1). It is a severe, uncommon disease with 1–2 cases per million people per year incidence, which is three times greater in the Far East. It is characterized by severe clinical sequelae and disproportionately affects children and young adults (2). Thanks to advancements in diagnosis and support-specific medicines [such as hematopoietic stem cell transplantation and immunosuppressive therapy (IST)], the prognosis has significantly improved recently, increasing the likelihood of recovery to up to 90%. The most prevalent type of SAA is acquired or immunological, and it is brought on by aberrant T-lymphocyte activation and hyperfunction, which results in the immune system destroying hematopoietic stem cells (HSCs) (2, 3). Transplantation of hematopoietic stem cells (HSCT) is the first-line treatment for SAA. There are now more viable options for SAA due to the availability of haploidentical donors and their effective use in transplantation. In fact, a lot of progress has been achieved in recent years with the use of haploidentical HSCT (haplo-HSCT) to treat SAA (4). HSCT or IST represents the first line of therapy depending on the possibility or otherwise of a fully suitable human leukocyte antigen (HLA) donor sibling (2, 5). Antithymocyte globulin (ATG) and cyclosporine A are components of the standard immunosuppressive treatment (IST) protocol (CsA). According to studies, horse ATG is superior to rabbit ATG for the treatment of children with aplastic anemia in terms of both short- and long-term response and survival (3, 6). However, malignant transformation or relapse is considerably common in patients receiving IST compared to those receiving HSCT from matched sibling donors (MSD); Around one-third of patients receiving horse ATG and CsA do not respond, 30% of responders experience relapse, and between 8% and 18% of patients experience clonal evolution (7). All SAA patients have significantly decreased levels of early progenitor and hematopoietic stem cells (8). Eltrombopag (ELT), an oral thrombopoietin receptor agonist, can also lead to clinically significant improvements in blood counts in nearly half of the patients with aplastic anemia refractory to immunosuppression (8), increasing bone marrow cellularity, CD34+ count, and progenitor cells through a direct effect on marrow stem cells (9). Numerous studies demonstrated its efficacy in patients with aplastic anemia refractory to immunosuppression. In fact, about 45% of patients experienced a hematological response to ELT alone, having not only higher platelet counts but also significant improvements in hemoglobin levels and neutrophil counts (8, 10). According to European Medical Agency (EMA), ELT is indicated in adults with acquired severe aplastic anemia who were unsuitable for HSCT or who had previously failed to respond to earlier immunosuppressive medication; in primary immune thrombocytopenia (ITP) in children aged one year and older who have not responded to other therapies for at least six months following diagnosis; for the treatment of thrombocytopenia in adult patients with chronic hepatitis C virus (HCV) infection, where the severity of the condition is the primary barrier to initiating or limiting the ability to maintain optimal interferon therapy, and in adult patients with SAA resistant to prior immunosuppressive therapy or heavily pretreated and ineligible for haematopoietic stem cell transplantation (11). In November 2018 the US Food and Drug Administration (FDA) has expanded the label for Eltrombopag to include first-line treatment for adults and children ≥2 years with SAA in combination with IST (12, 13). The approval followed a study initiated by the National Institutes of Health (NIH) that showed significant improvement in both the overall response rate (ORR) and complete response (CR) compared to a historical cohort of IST (2, 14). However, the effects of ELT on pediatric patients with SAA remain controversial and limited. Data from adults showed that adding ELT resulted in considerable increases in response rates to >80%, nevertheless, the same outcome did not occur in children (5, 15). Additionally, Jin et al. discovered that among Chinese adult patients with SAA treated with IST plus ELT as opposed to those without ELT, the complete ORR of hematologic response was higher (6). While the addition of ELT to IST for pediatric patients with AA did not improve the ORR at 6 months (3). ELT was generally well tolerated, with a low rate of grade 3–4 serious adverse events (5%) that was similar to that observed in the placebo group (16). The present systematic review aims to evaluate the available evidence on the efficacy and safety of ELT added to IST for the treatment of SAA in the pediatric population.

## 2. Methods

### 2.1. Search strategy and selection criteria

This study was conducted following the PRISMA (Preferred Reports of Systematic Review and Meta-Analysis) guidelines (17) to perform the search, extraction, reporting and presentation of standardized data and was registered in PROSPERO (CRD42022325859). Three search engines were used to conduct a systematic review of clinical studies of ELT in children with SAA published up to the 20th of December 2022: PubMed, Web of Science, and Embase. Two researchers (AM and MM) conducted the search technique after reading the scientific literature, which is displayed in supplemental Table 1. The full-text document was read if the structured abstract was suitable or raised concerns. Just the papers that matched the inclusion criteria from the materials that were fully read were accepted for systematic review. Inclusion criteria were identified by a researcher (MM) who also initiated the review of the titles and abstracts of all retrieved studies. These criteria were then evaluated by another researcher who independently reviewed the papers (AM). Whenever there were any disagreements between the two investigators, a third investigator (FR) was consulted to reach a conclusion. A population/participant, intervention, comparator/control, outcome, study design framework, known as PICOS, was developed. Population: pediatric patients younger than eighteen years of age, except for a portion of the adult population included in large adult pediatric studies, diagnosed with SAA, never treated previously, were included; All patients with congenital bone marrow failure syndromes were excluded. Intervention: first-line treatment with ELT in addition to IST; Comparator: standard IST (if reported) or none. Outcome: Efficacy of adding ELT to IST in the pediatric population, highlighting whether the improvement in the complete and partial response rate of the hematological profile can be comparable to what was already observed in the adult population, and describing any adverse events occurring after ELT treatment. Study design: clinical trials, observational studies, and case report/series. Chapter books, preclinical research, reviews, letters, posters, comments, editorials, articles in languages other than English, and recommendations were excluded.

**Table 1:** Search strategy.

**PubMed**
[(SEVERE APLASTIC ANEMIA) OR (ACQUIRED APLASTIC ANEMIA)] AND (ELTROMBOPAG) AND (CHILDREN)
**Web of Science**
((ALL = (severe aplastic anemia)) OR ALL = (acquired aplastic anemia)) AND ALL = (eltrombopag) AND ALL = (children)
**Embase**
([ALL = (severe aplastic anemia)] OR ALL = (acquired aplastic anemia)) AND ALL = (eltrombopag) AND ALL = (children)

### 2.2. Data extraction

The following information was taken from each chosen article: year of publication, type of study, number of patients, number of males and females, age, dose of ELT/IST, comparison (if reported), follow-up, and study outcome, adverse effects.

## 3. Result

### 3.1. Study selection and characteristics

**Figure 1:**
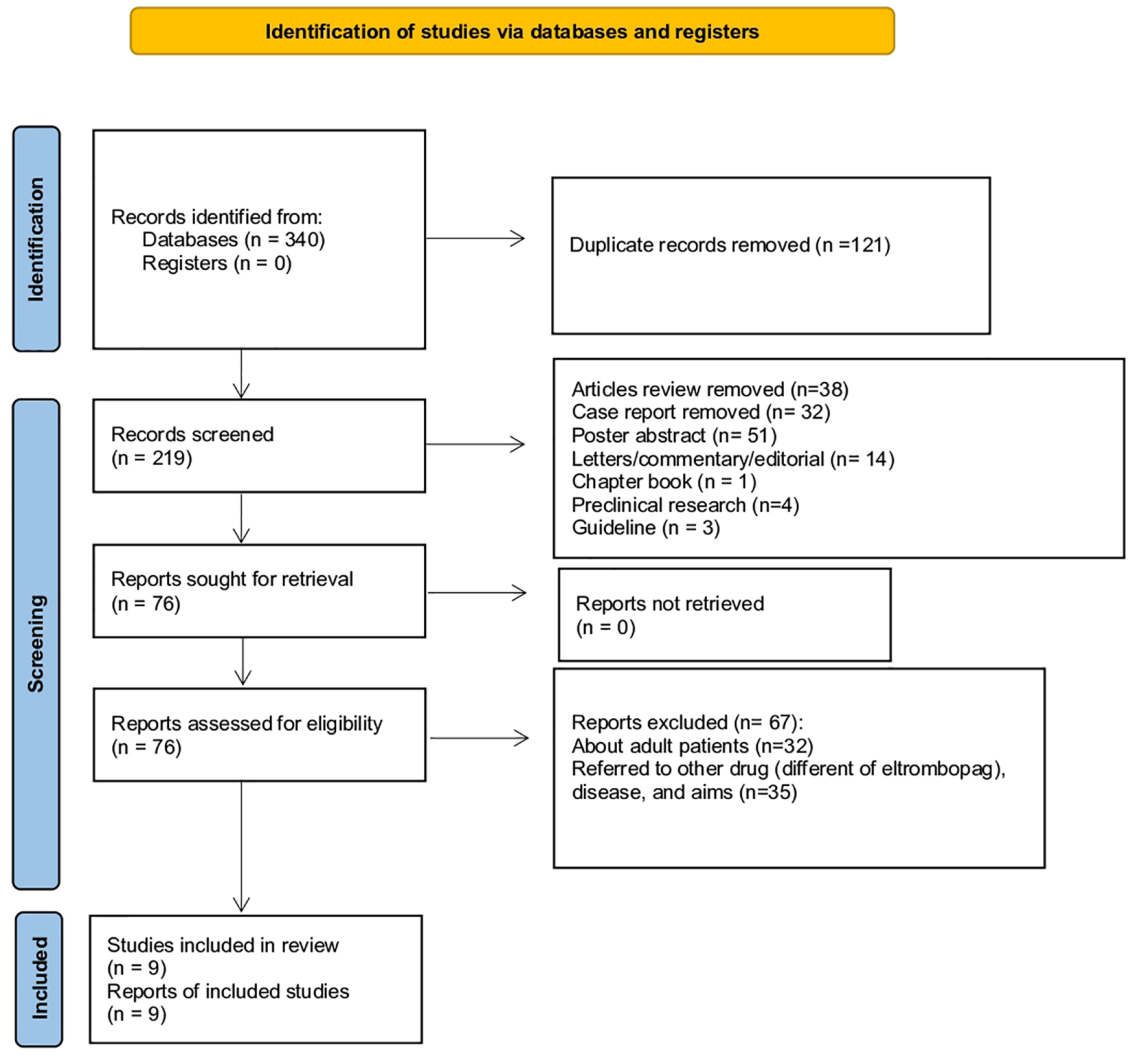
PRISMA flowchart of the identification and inclusion of systematic review.

In this systemic review, we retrieved a total of 340 articles, of which 209 from Embase, 62 from Web of Science, and 69 from PubMed. A total of 121 articles were deleted because duplicates, thus 219 articles were screened for inclusion/exclusion criteria. Specifically, we excluded records because they were: review/meta-analysis (*N* = 38), case report (*N* = 32), poster abstract (*N* = 51), letters/commentary/editorial (*N* = 14), chapter book (*N* = 1), preclinical research (*N* = 4), and guideline (*N* = 3). A total of 45 records were observational studies excluded because conducted in adult patients (*N* = 26) and/or correlated to other drugs (different from ELT) or disease (*N* = 19). A total of 22 records were clinical trials excluded because conducted in adult patients (*N* = 6) or related to other drugs (different of eltrombopag), diseases, or aims (*N* = 16). As a result, a total of 9 records were identified, of which 4 clinical trials, 3 observational studies, and 2 case series (Figure 1). The general characteristics of clinical studies are shown in Table 2.

**Table 2:** General characteristics of the included studies.

Authors	Publication year	Study design	Population (M/F)	Age	IST dose	ELT dose	% ORR, PR, CR, NR, Relapse, 3 months IST	% ORR, PR,CR NR, Relapse, 6 months IST	% ORR, PR, CR, NR, Relaps, 3 months IST + ELT	% ORR, PR,CR NR, Relapse, 6 months IST + ELT	Clonal Evolution and Bone Marrow Evaluation
Fang M. et al.	2021	Observational study	18 IST + ELT 39 IST	≤16 yo (1.3–13.5)	pATG (30 mg/kg/die) CsA 3–5 mg/kg/day	1 mg/kg/day (maximum dose 50 mg/day)	ORR 56.4% CR 12.8% PR 43.5% NR 43.5% Relaps 0	ORR 69.2% CR 17.9% PR 51.2% NR 30.7% Relaps 0	ORR 77.7% CR 22.2% PR 55.5% NR 22.2% Relaps 0	ORR 94.4% CR 50% PR 44.4% NR 0 Relaps 5.5%	None
Jie et al.	2021	Observational study	14 IST + ELT	<18 yo	rATG 3,3-3,5 mg/kg/day CsA 5 mg/kg/day	75 mg/day in >6 yo;2.5 mg/kg/day in 2–5 yo	ORR 64.3% CR 10.7%	ORR 67.8% CR 21.4%	ORR 35.7% CR 7.1%	ORR 78.5% CR 57.1%	None at 6 months; 35% at 12 months; 42% at 24 months. None increased marrow fibrosis.
Lesmana H. et al.	2021	Observational study	9 IST + ELT 16 IST	<18 yo	hATG 40 mg/kg/day; CsA 5 mg/kg/day	25 mg/day in <6 yo; 50 mg/day 6–8 yo; max 150 mg/day	NR	ORR 71% CR 29% NR	NR	ORR 100% CR 29% NR 0	No clonal evolution or bone marrow alteration.
Filippidou M. et al.	2020	Case series	11 IST + ELT	<18 yo (2.7–15.5)	NR	From 25 to 150 mg/day	NR	NR	PR 73% NR 18% CR 9%	NR	None None increased marrow fibrosis
Min-Yu Su et al.	2021	Case series	8 IST + ELT	<16 yo (3–16)	NR	From 25 mg/day	NR	NR	CR 25% PR 50% NR 25%	NR	NR
Groarke E.M. et al.	2020	Clinical trial	40 IST + ELT 87 IST	<18 yo	hATG 40 mg/kg; CSA 3 mg/kg	150 mg/day in ≥12 yo, 75 mg/day in 6–11 yo; 2.5 mg/kg in 2–5 yo	ORR 63% CR 14% PR 49% NR 33%	ORR 72% CR 23% PR 49% NR 22%	ORR 68% CR 23% PR 45% NR 23%	ORR 70% CR 30% PR 40% NR 10%	13%
Townsley D.M et al.	2017	Clinical trial	92 IST + ELT	<18 yo 18–64 yo ≥65 yo		150 mg/day in 12 yo; 75 mg/day in 6–11 yo; 2.5 mg/kg/day in 2–5 yo	NR	NR	ORR 80% PR 50% CR 30%	ORR 87% PR 48% CR 39%	7% at two years
de Latour R. P. et al.	2022	Clinical Trial	101 IST 96 IST + ELT	15–81 yo	hATG 40 mg/kg/day; CSA 5 mg/kg/day	150 mg/day	ORR 31% PR 21% CR 10% NR 69%	ORR 41% PR 21% CR 20% NR 59%	ORR 59% PR 38% CR 22% NR 41%	ORR 68% PR 37% CR 32% NR 32%	No evidence of myelodysplastic syndrome.
Goronkova O. et al.	2022	Clinical Trial	49 IST 49 IST + ELT	2–18 yo	hATG 40 mg/kg/day; CSA 5 mg/kg/day	2 mg/kg/day	*ORR 53% PR 41% CR 12% NR 47%	ORR 51% PR 33% CR 18% NR 45% Relaps 2%	*ORR 65% PR 35% CR 31% NR 33%	ORR 55% PR 20% CR 35% NR 33% Relaps 10%	No evidence of myelodysplastic syndrome or PNH status.

ORR, overall response rates; CR, Complete response; PR, Partial response; NR, no responders. NR, not reported; *follow up 4 months.

### 3.2. Observational studies

Three retrospective observational studies involved a total of 96 patients, male and female, aged ≤18 years who were diagnosed with SAA between January 2012 and January 2020 (3, 6, 18). All three articles defined SAA by a bone marrow cellularity <30% and at least 2 of the following criteria, including absolute neutrophil count (ANC) <0.5 × 109/L, pre-transfusion platelet count <20 × 109/L, and pre-transfusion reticulocyte count <20 × 109/L. All three studies included patients with newly diagnosed, untreated SAA; the study excluded those with known causes of secondary aplastic anemia, congenital aplastic anemia, substantial impairment of heart, liver, or kidney function, severe uncontrolled infection, or any other uncontrolled condition. The first single-center retrospective cohort study enrolled a total of 57 patients with a newly diagnosed SAA. 39 patients received CsA at a starting dose of 3–5 mg/kg/day from day 8 until the end of the 2-year period, which was then fine-tuned and adjusted to maintain a trough blood concentration between 200 and 250 ng/ml and intravenous infusion of porcine ATG (30 mg/kg/day) that was continued for 18 h each day for 5 consecutive days. Eighteen patients were treated with IST and ELT (from day eight to the end of the 6-month period) with the initial dose of 1 mg/kg/day (max dose 50 mg/day) that was then continued for six months, modified in accordance with the outcomes of complete blood counts. From this study emerged that when ELT was added to IST, there was a statistically significant increase in the ORR to 94.4% and CR to 50% (3). Thirty-six individuals had fever, while 15 patients experienced serum sickness, which all resolved after symptomatic treatment. The main adverse event of ELT was the elevation in bilirubin levels, accounting for 66.6% of patients in the ELT group vs. 20.5% of patients in the IST group (*P* < 0.05). Additional side effects included nausea, vomiting, and dizziness, all of which went away spontaneously or after treatment. No patient suspended ELT due to adverse events (3) (Adverse events related to the different studies are described in Table 3). The second retrospective study enrolled 14 patients treated with rabbit ATG at a dosage of 3.3–3.5 mg/kg/day intravenously for 5 days; To keep serum trough concentration at around 100–200 mg/dl, administer CsA at a dose of 5 mg/kg/day orally commencing on day 1; administer ELT at a dose of 75 mg daily for patients older than 6 years old and 2.5 mg/kg body weight/day for children between 2 and 5 years old (6). CR and ORR were 64.3% and 78.6% at 6 months, respectively. In responders, the survival rate was 100% and no relapse occurred. In this study, no patient discontinued the treatment with ELT due to adverse events. Indirect bilirubin elevation and jaundice were the most common adverse events (64.3%), while the transient increase in liver enzyme levels was observed in only 21.4% of patients. The aforementioned abnormal laboratory parameters were self-resolved or disappeared after ELT discontinuation. No patient developed thromboembolic events, cataracts, myelofibrosis, or rashes (6). The third retrospective study involved 16 patients treated with IST and 9 patients treated with the combination IST and ELT. All patients were treated with horse ATG 40 mg/kg every 24 h for 4 days and CsA at 5 mg/kg/day with a dose adjusted to maintain trough levels of 170–270 ng/ml. In the group treated with ELT, this drug was added within 1 week from the IST regimen in seven patients, while two patients received ELT in a refractory setting after IST failure. ELT was used at 25 mg/day in patients aged under 6 years old and 50 mg/day in patients aged 6–18 years and was titrated to a dose of 150 mg/day, if tolerated (18). In the IST arm, the ORR was 71% and 100% and the CR was 29% and 58% at 6 and 12 months, respectively. Seven patients receiving the combination IST-ELT showed an ORR at 6 and 12 months, with two of them (29%) also achieving CR at 6 and 12 months. ELT had no effect on two patients who had previously failed IST. In both cohorts, there was an acceptable safety profile. Specifically, the risk of infectious complications (grade ≥3), such as febrile neutropenia and confirmed infections, were common in both cohorts, occurring in 81% of patients in IST group and in 44% of patients in IST-ELT group. Renal failure was more common in patients receiving the combination of IST and ELT than IST alone (67% vs. 6%; *P* = 0.003), although none of these events was categorized as graded ≥3. Only one patient in the IST-ELT group had severe transaminitis, which decreased after dose reduction. No cutaneous toxicity was found in the eltrombopag group (18).

**Table 3:** Adverse events attributed to eltrombopag.

Study	Adverse Events
Lesmana H. et al.	Febrile neutropenia; infection 44%;
	Renal insufficiency 67%
	Transaminitis 11% (1 pz)
Fang M. et al.	Hyperbilirubinemia 66.6%
Jie et al.	Indirect bilirubin elevation and jaundice 64.3%, 9 patients
	Transient elevations in liver enzyme 21.4%, 3 patientes.
Filippidou M. et al.	None
Min-Yu Su et al.	Hyperbilirubinemia (range: 1e4 mg/dl) 75%,6 pz
	Bone infarct of bilateral femoral and tibial shaft 12.5%, 1 pz
	Skin rash 12%, 1 pz
Townsley D.M et al.	Skin 3%
	Abdominal pain 2%
	Liver test abnormality 18%
	Blood bilirubin increased 13%
Groarke E.M. et al.	Skin 5% 2 pz
	Blood bilirubin increased 7.5% 3 pz
	Liver function abnormalities 40%1 0 pz
de Latour R. P. et al.	Elevated liver enzyme levels in 4 pz;
	Slight increase in reticulin deposition in the bone marrow in 2 pz.
Goronkova O. et al.	Liver test abnormalities: grade 1–2 59%; grade 3–4 61%.
	Skin hyperpigmentation: 12%.
	Hyperbilirubinemia: 45%.
	Increased alanine aminotransferase levels 45%.
	Increased aspartate aminotransferase levels 31%.
	Infectious complications/febrile neutropenia 80%

### 3.3. Clinical trials

The investigator-initiated, nonrandomized, historically controlled phase 1–2 clinical trial, published by Townsley et al. in the New England Journal, enrolled 92 consecutive patients aged 2 years or older to evaluate the IST plus ELT. It enrolled three cohorts with different ELT regimen, start time, and length (ELT was provided to cohort 1 from day 14 to six months, cohort 2 from day fourteen to three months, and cohort three from day one to six months.). The cohorts were compared to a historical cohort of 102 patients who received CsA and horse ATG while taking part in two of the most recent clinical studies. ELT was administered at a dose of 2.5 mg/kg/day in patients aged 2–5 years, at a dose of 75 mg/day in patients aged 6–11 years, and at a dose of 150 mg/day in patients ≥12 years. For all patients, the median follow-up was 703 days (23 months; range: 84–1,422), and for patients who were still alive, it was 713 days (24 months; range: 194–1,422). In cohorts 1, 2, and 3, the CR at 6 months was 33%, 26%, and 58%, respectively, while the ORRs were 80%, 87%, and 94%. Both CR and ORR were higher in the combination group than in the historical cohort (CR was 10% and ORR was 66%). The survival rate was 97% at a 2 years of median follow-up; one death was from a non-hematologic cause. The amount of CD34+ cells, the frequency of early hematopoietic progenitors, and bone marrow cellularity all showed significant increases. Relapse and clonal evolution rates were similar to that observed in the historical cohort. Liver abnormalities, despite frequent, were often transient and not associated with alteration of ELT therapy. Seven patients discontinued ELT within the first 2 weeks due to transient increased liver enzyme levels, and two patients due to serious skin rashes. Serious skin rashes were observed in one patient of cohort 2 (at 6 weeks) and one patient of cohort 3 (at 4 weeks), and were associated with fever and oral pain resulting also in hospitalization and glucocorticoids treatment. The other two serious events attributed to ELT were abdominal pain. Adverse events not attributed to ELT by the investigators were neutropenic infections and toxic effects from ATG and CsA. One patient died 3 months after treatment for a non-hematologic disease (paraneoplastic encephalopathy), which was attributed to thymoma presented before study entry (8). The second was a subgroup analysis of the above clinical trial (8), involving 40 children with a treatment-naïve SAA and a comparison arm represented by a pediatric historical cohort of 87 children treated with standard IST (2). The eltrombopag group had a median follow-up of 1,432 days, while the control group of 2,409 days. No significant difference in the ORR (ORR 70% in ELT cohort, 72% in historical cohort, *P* = 0.78) or CR (30% with ELT, 23% with historical control, *P* = 0.42) at 6 months was observed. Response rates were lower in younger children than adolescents. Indeed, the ORR was 63% for ELT compared with 78% for IST (*P* = 0.29) in children. Adolescents instead had an ORR of 75% with ELT compared to 67% with IST (*P* = 0.48), respectively. The trend towards relapse was higher and event-free survival was significantly lower in children treated with ELT compared to IST. The safety profile of eltrombopag was good in children; no patients experienced severe sequelae following eltrombopag administration or died. Three serious adverse events (SAEs) occurred in the pediatric eltrombopag group that required drug withdrawal; two cutaneous rashes (grade 2 and 3), and one hyperbilirubinemia (grade 3). Reversible liver function abnormalities occurred in 10 patients treated with eltrombopag, which were reported as grade 2–3 increase in bilirubin, aspartate aminotransferase and alanine transaminase (2). A prospective, open-label, multicenter, randomized study by Goronkova et al. that enrolled 98 pediatric patients, aged 2 to 18 years, with naive SAA and no matching donor siblings who were randomized to receive horse ATG and CsA with (*n* = 49) or without (*n* = 49) ELT. In the IST group, patients received horse ATG 40 mg/kg/day for 4 days and 5 mg/kg/day of CsA starting on day 1. After achieving the hematological response plateau, CsA was continued for at least 18 months before being gradually reduced by 5%–10% each month. In the ELT and IST group, patients also received eltrombopag at a dose of 2 mg/kg/day starting on day 1 of horse ATG. ELT was administered for at least 120 days; if no partial response (PR) was observed at 4 months, ELT was stopped, and patients received second-line therapy; if at least a stable PR was observed, the ELT dose was decreased and withdrawn over an eight-week period. The ORR at 4 months served as the main endpoint. The ORR in all patients was comparable between the ELT-IST and IST groups (65% vs. 53%), however the CR rate in the ELT + IST group was much greater (31% vs. 12%). No significant differences in the ELT-IST and IST groups were observed in 3-year overall survival (89% vs. 91%, *P* = 0.673) or 3-year event-free survival (53% vs. 41%, *P* = 0.326). This study failed to achieve a 20% target benefit in ORR at 4 months in favor of ELT and IST. The ELT-IST group's CR rate was found to be significantly greater than the IST group's (31% vs. 12%, *P* = 0.027). The first randomized evidence of the benefit of adding ELT to IST in SAA children who have not received treatment is this considerable improvement in CR with ELT. Significantly, when ELT was combined with IST as opposed to IST alone, neutrophil recovery was accelerated and the ANC was considerably higher at 2, 3, and 4 months following the start of treatment. The proportion of patients who received G-CSF was comparable in both groups. Between the two groups, there were no discernible differences in overall survival. Both groups' safety profiles were acceptable. The only ELT-related adverse events leading to ELT treatment interruption or discontinuation were liver test abnormalities, as expected. No cases of clonal evolution to myelodysplastic syndrome, acute myeloid leukemia, or haemolytic paroxysmal nocturnal hemoglobinuria (PNH) occurred in this study at a median follow-up of 2.3 years (7). In the prospective, open-label, multicenter, randomized, phase 3 study conducted by de Latour et al., the efficacy and safety of ELT with or without horse ATG and CsA as first-line therapy were compared in patients with a history of SAA. From July 2015 to April 2019, a total of 197 patients of 15 years of age and older were assigned to receive either IST (First Group, 101 patients) or IST plus ELT (Second Group, 96 patients). First group patients received horse ATG at a dose of 40 mg/kg/day for 4 consecutive days and oral CsA at a dose of 5 mg/kg/day from day 1 for a minimum of 12 months; Second group patients received IST plus orally administered ELT at a dose of 150 mg daily from day 14 for up to 6 months or for up to 3 months in patients who had a complete response at 3 months. Patients in the second group who after 3 months exhibited a partial response (described below) continued to receive ELT for an additional 6 months; The median follow-up among patients in both groups was 24 months. At three months, 10% of patients in the first group and 22% of patients in the second group had a full response. The ORR was 41% in the first group and 68% in the second group after six months. The 3-month ORR was lower in the first group (31%) than in second group (59%). At 24 months, the cumulative incidence of PNH was 7% in the first group and 1% in the second group. Eighteen months after the response, there was no discernible difference in the cumulative incidence of recurrence between the first (11%; 95% CI, 2 to 20) and second (19%; 95% CI, from 9 to 29) groups. Age was the only factor linked to lower overall survival and recurrence risk. After a follow-up of 2 years, one patient in the first group and two patients in the second group both acquired a karyotypic anomaly categorized as a myelodysplastic syndrome; event-free survival was 34% and 46%, respectively. In this study, the 3-month complete and global hematologic responses were significantly better with the addition of ELT to standard IST than with IST alone, and the quality and speed of hematologic recovery were also better with the addition of ELT. ELT appears to assist hematopoiesis, regardless of its molecular processes, buying time for immunosuppression to stop the immune system's attack on hematopoietic stem cells. In this trial, the combination IST and ELT resulted in shorter median times to first response and full response than IST alone; These quicker reaction times demonstrated the ELT group's earlier attainment of red blood cell and platelet transfusion independence. At six months, partial response required transfusion independence, and the overall response rate increased from 41% to 68%. In contrast to horse ATG plus CsA alone, this prospective randomized study showed that adding ELT to horse ATG plus CsA was advantageous for patients with SAA. ELT was added, which resulted in a quicker and higher-quality response without any discernible harmful consequences (19).

### 3.4. Case series

A case series by Su et al. involved 8 pediatric patients with SAA treated with ELT. Three patients received IST prior to ELT for at least 3 months. Five patients received IST and ELT concomitantly (ELT started within 7 days before or after IST). The dose of ELT started at 25 mg/day and was adjusted according to the clinical picture. No obvious side effects occurred during the duration of IST with ELT. A commonly observed adverse event was hyperbilirubinemia, which was observed in 6 patients (75%). One patient experienced a cutaneous reaction that gradually disappeared. Only one case of bilateral bone infarction of the femur and tibial diaphysis was recorded, which occurred after the use of ELT for 4 weeks. The PR rate was at least 80% at 3 months. This seems better than STI alone, which showed a response rate of about 42% at 3 months. During the most recent follow-up, just 1 patient still required transfusions, and 3 of them were transfusion free. One of the 5 patients had a relapse of the disease and underwent TCS. The mean duration of eltrombopag use was 12.9 months (20). The other case series considered 11 pediatric patients who received ELT in addition to standard IST. ELT was started from day 0 and within 2 months after the onset of IST. The safety profile for ELT was satisfactory, and no significant adverse events were noted. Six months after stopping the CsA treatment, one patient developed moderate thrombocytopenia, necessitating the return of ELT. With the early addition of ELT to IST, we saw a substantial increase in outcomes because patients who got ELT commencing on day 0 of ATG responded better and faster in CBC, notably in platelet count, showing a synergistic impact of ELT with ATG. No patient had any significant adverse effects or signs of bone marrow clonality or fibrosis at the time of the most recent analysis, which followed a lengthy course of eltrombopag therapy (up to 36 months). Long-term overall survival was 90%–9% and event-free survival was 72%–7%. After extended observation time, the long-term overall remission rate among patients who had an initial response was 89%, suggesting a durable response that was at least equivalent to rates anticipated for pediatric patients receiving IST. The response rate in pediatric IST patients is higher than the reported rate of 60%–70% in historical adult cohorts among patients who achieved CR with IST. Three patients who took ELT simultaneously with ATG experienced quicker CBC recovery and transfusion independence within the first three months following ATG compared to those who delayed medication beginning. Eltrombopag was taken on average once a day at a dose of 75 (25–150) mg for a median time of 103 (1–27) months (9).

## 4. Discussion

Infancy-onset aplastic anemia is typically acquired and brought on by the immune system's destruction of hematopoietic progenitor cells. The preferred treatment for kids ineligible for MSD-HSCT is the IST with horse hATG + CsA, with an initial ORR of 70%–80% (6). Responses to IST are good, but there are still worries about clonal evolution and recurrence, especially in youngsters who will benefit the most over time (3). Generally, children have better IST response rates than adults and tolerate more intensive IST regimens (3). According to EMA, ELT is recommended for treating adult individuals with primary immune thrombocytopenia (ITP) who have failed previous therapies; is indicated for the treatment of pediatric patients aged one year and older who have primary immune thrombocytopenia (ITP) that has not responded to alternative treatments for at least six months after diagnosis; in adult patients with chronic hepatitis C virus (HCV) infection for the treatment of thrombocytopenia, where the severity of the condition is the primary barrier to initiating or limiting the ability to maintain optimal interferon therapy, and in adult patients with SAA resistant to prior immunosuppressive therapy or heavily pretreated and ineligible for hematopoietic stem cell transplantation (11). The combination of ELT and IST was approved by the US Food and Drug Administration (FDA) for adults and children ≥2 years of age as a first-line treatment for SAA (12, 13). Furthermore, recently, ELT has demonstrated in numerous studies several activities, such as immunomodulation, acting as a mediator of macrophage polarization from the proinflammatory M1 to the anti-inflammatory M2 phenotype, ameliorating the inflammatory and immune-compromised profile, and its effects on osteoclast activity (21, 22). Recent studies have demonstrated that ELT may be effective in patients with aplastic anemia refractory to immunosuppression (5). Even after drug termination, the addition of ELT to the IST aids in the quick and robust recovery of blood counts and the restoration of trilineage hematopoiesis in adult patients with SAA (9). The pediatric studies covered by our systematic search show conflicting results. All three observational studies demonstrated that the addition of ELT to the IST contributes to rapid and robust recovery of blood cell counts and restoration of trilineage hematopoiesis (3, 6, 18). In Fang's study, after 6 months of treatment, the ORR in the IST group was 69.2%, significantly lower than the ORR of the IST-ELT group 94.4%, and the ORR was only 17.9%, compared with 50% in the IST-ELT group; no statistical difference was found between the 3-month response rates for the two groups (3). In the Jie et al. trial, the ORR at 6 months following IST with ELT was also noticeably greater than in the historical group. Overall long-term survival was 100%, and 78.6% of patients had event-free survival (6). In Lesmana's work, among IST patients, 29% of patients achieved CR and 43% achieved PR for an ORR of 71% after 6 months. All seven patients in the IST-ELT group who received IST and ELT at the same time responded, with 29% reaching CR. At 6-month ORR between IST and IST-ELT showed no change. 25% of IST patients had treatment failure. In the IST-ELT study, no treatment failures were observed at 6 months and all maintained their response for an ORR of 100% at 1 year. Consequently, the 1-year overall survival rate in IST was 88% and in IST-ELT it was 100%. The 1-year EFS rate in IST was 75% and in IST-ELT it was 100% (18). Among clinical trials Townley, Goronkova and de Latour studies are in favor of adding ELT, while the Groarke study seems ineffective (2, 7, 8, 19). In the clinical trial by de Latour et al., the quality and speed of hematologic recovery were also improved with the addition of ELT, without excess toxic effects, and the 3-month complete and global hematologic responses were significantly better with the addition of ELT to standard therapy than standard therapy alone (10% IST vs. 22% IST-ELT). The ORR at 6 months with IST plus ELT was also significantly (79%), better than with IST alone (66%). At 12 months, the CR rate was 33% in IST group and 52% in IST-ELT group. The 2-year overall survival was similar in IST group (85%) and IST-ELT group (90%). In this study, the median times to first response and time to CR were shorter with IST plus ELT than IST alone. The quicker hematologic recovery observed may result in fewer patients needing to switch to early hematopoietic stem cell transplantation, even though the difference in response rate gradually decreases over time (19). According to de Latour, less severe aplastic anemia and younger age were associated with a better response. However, ELT significantly increased event-free survival from 34% to 46% at 2 years and induced a more rapidly higher quality response, but it should be noted that the vast majority of enrolled patients were adults, except for 9 teenagers aged 15–18 (19). Data presented in the nonrandomized subgroup analysis of Groarke et al. do not support adding ELT to IST for untreated SAA in children. There was no significant difference in either the ORR or CR rate. Compared to the IST group, which had an ORR of 63% at 3 months and 72% at 6 months, the ELT group's ORR was 68% at 3 months and 70% at 6 months. At one year, there was no discernible difference in response rates between the IST and ELT groups. Adolescents achieved a CR rate of 46% with ELT compared to 21% with IST, and overall responses of 75% vs. 67% respectively. Recurrence was higher and EFS significantly lower in the ELT group (2). The study by Goronkova et al. failed to achieve a 20% target advantage in ORR at 4 months in favor of ELT-IST, according to the pediatric study by Goarke et al. All patients had similar ORRs in the ELT-IST and IST groups (65% vs. 53%), however, the CR rate in the ELT-IST group was much greater (31% vs. 12%). At 6 months after the crossover, 61% of initial without ELT patients achieved a response compared to 17% of initial ELT patients. In Goronkova's investigation, it was discovered that the ELT-IST group had a much greater CR rate than the IST group did. This significant improvement in CR with ELT was the first randomized evidence of the benefit of adding ELT to IST in treatment-naïve SAA children. In conclusion, this randomized study's findings justify the inclusion of ELT to IST as the initial course of therapy for young patients with SAA. By considerably raising the ANC and CR rate, this combination expedites and enhances the hematological response. Overall survival did not differ significantly between the two groups, which is consistent with the aforementioned research. Relapse and EFS rates were comparable across the two therapy groups (7). According to Townsley, ELT appears to significantly boost the frequency, rapidity, and robustness of hematologic recovery in patients with SAA when added to the current recommended regimen of immunosuppression (8). The response rate was also greater than anticipated with conventional immunosuppression: in the present research, it was 87%, against 66% in our previous cohort. Over 95% of patients survived after a median follow-up of two years. The median follow-up in the current study was 2 years, although the median duration to clonal development with immunosuppressive medication is 4–6 years. All studies agree on the need for a longer follow-up to assess response durability and clonal evolution final risk (8). The safety profile in all studies was acceptable. The most frequently recorded adverse events were hyperbilirubinemia, increased liver enzymes, and skin rash. These regressed immediately after suspension or discontinuation of treatment. More rarely we have observed febrile neutropenia, renal insufficiency, and bone infarction. Immunosuppressive treatment had a median time to clonal evolution of 4–6 years, which is longer than the median follow-up of 2 years across the various trials (7). The role of the early addition of ELT in enhancing clonal evolution is still unclear, and much longer follow-up is needed to shed light on this issue. In conclusion, based on the studies we reviewed, eltrombopag definitely seems to support hematopoiesis, buying immunosuppression time to curb the immune attack on hematopoietic stem cells, and accelerates and improves the quality of hematological response, significantly increasing the rate of ANC and CR, without excessive toxic effects, although not all results obtained from our studies support the addition of ELT to IST in the first-line treatment of pediatric patients with SAA. However, not all studies agree, and the efficacy appears to be better in the adult population than in the pediatric population. These findings do not modify the current algorithm of SAA treatment in children; HSCT is currently advised as the first-line treatment for children with an HLA-identical sibling and immunosuppressive therapy in kids without a sibling (23). The results of ongoing studies and the experience accumulated at pediatric hematology centers will be important to definitively address the role of thrombopoietin mimetics in children.

## 5. Limitations

The heterogeneity of the data provided by the studies, especially relating to the dosage and timing of non-standardized administration of ELT, the type of population, mixed in some studies and small size, the limited follow-up, did not allow us to complete the study with meta-analysis.

## Data Availability

The original contributions presented in the study are included in the article/Supplementary Material, further inquiries can be directed to the corresponding author.
